# Arthroscopic reconstruction of the Ligamentum Teres: a case series in four patients with connective tissue disorders and generalized ligamentous laxity

**DOI:** 10.1093/jhps/hnw016

**Published:** 2016-06-26

**Authors:** Sivashankar Chandrasekaran, Timothy J. Martin, Mary R. Close, Carlos Suarez-Ahedo, Parth Lodhia, Benjamin G. Domb

**Affiliations:** Hinsdale Orthopaedics, American Hip Institute, 1010 Executive Court, Suite 250, Westmont, IL 60559, USA

## Abstract

This study presents the results of four Ligamentum Teres (LT) reconstruction procedures for hip instability with an average of 21.4 months follow-up (range 16.4–27.8). The indication for reconstruction was patients who complained of hip instability (hip giving way on gait or activities of daily living) on a background of a connective tissue disorder and generalized ligamentous laxity. The following data were recorded: age, sex, body mass index, hip range of motion, impingement signs, acetabular coverage (lateral center edge angle and acetabular inclination), acetabular retroversion (ischial spine sign and a crossover sign), femoral alpha angles and femoral neck shaft angles. Four patient recorded outcomes (PROs) were collected at 3 months, 12 months and 24 months. Three patients were female. Three out of four procedures had an improvement in PROs. One patient with bilateral procedures had an improvement in PROs on one side at 1 year but a failure of the graft on the contralateral side. There were no complications reported with the technique. LT reconstruction and concomitant capsular plication in this case series is associated with an improvement in outcomes in three out of four of the patients with hip instability associated with a full thickness tear of the LT and who presented with hip instability on a background of generalized ligamentous laxity and a connective tissue disorder. However, the physical examination, radiographic and intra-operative findings which may help predict who would benefit from LT reconstruction require further investigation.

## INTRODUCTION

Bony and soft tissue structures contribute to the stability of the hip joint. The soft tissue components of stability consist of the labrum, capsuloligamentous complex and ligamentum teres (LT) [1]. About 60% less force is required to distract the hip in the presence of a labral tear, supporting the concept of the importance of the labrum in hip stability [2, 3]. The importance of the capsuloligametous complex to hip instability is most noted after reports in the literature of hip instability or dislocation following hip arthroscopy in which a capsulotomy/capsulectomy was performed without closure [4–7]. In the adult, the LT was theorized to have limited biomechanical or vascular importance and hence was routinely sacrificed during procedures involving open surgical hip dislocation [8–10]. However, a recent review on the LT's increasing importance by O’Donnell *et al.* has highlighted that the LT has an important role in hip proprioception and mechanics [11]. Martin *et al*. used a string model to assess the excursion of the LT during hip movements. They reported that the LT may contribute to hip stability particularly in external rotation in flexion and internal rotation in extension [12]. In addition, they reported that in patients with inferior acetabular insufficiency or generalized ligamentous laxity, complete LT ruptures may result in instability during squatting and crossing one leg behind the other [12]. Similarly, Kivlan *et al.* [13] used human cadavers to demonstrate that when the human hip moves into flexion-abduction, the LT moves into a position that provides anterior and inferior stabilization of the hip. Furthermore, the prevalence of LT tears at arthroscopy ranges from 5 to 51%, raising the possibility that it may be a potential pain generator in these patients [14–16].

The optimal management of LT tears is not been clearly defined [17]. Debridement of selected fibers in patients with pain rather than instability has had favorable outcomes [18, 19]. Others have advocated LT reconstruction may help address pain in patients who present with instability [20]. Patients with predominantly instability symptoms are more likely to be female, ligamentous lax and have an increased range of motion of the hip [21]. Hip instability may manifest as groin pain and/or painful clunking or clicking of the hip that may be exacerbated by extension and external rotation activities of the hip [22]. Osseous anatomy may be normal or abnormal in these patients and treatment relies on stabilizing the secondary soft tissue restraints such as the labrum and capsule in addition to osseous corrections [23]. Van Arkel *et al.* conducted a biomechanical study which showed that the LT as a secondary restraint to hip stability in high flexion, adduction and external rotation with the primary restraints being the lateral arm of the iliofemoral ligament and the ischiofemoral ligament [24]. Therefore, in patients with instability symptoms on a background of connective tissue disorders or generalized ligamentous laxity reconstruction of a torn LT may be an important adjunct to restoration of capsular stability once bony morphology was determined as normal without any gross femur or acetabulum dysplastic characteristics [25]. Simpson *et al.* [20] were the first to report on the technique of LT reconstruction and Philippon *et al.* [26] have the largest series of four patients in whom the majority reported improved outcomes with reconstruction. Amenabar *et al.* [27] also reported improved patient outcomes with LT reconstruction. The purpose of this study is to report on the outcomes in three patients in whom four reconstruction procedures were performed and report indications and early clinical experience. These patients presented with hip instability on a background of ligamentous laxity with a known or probable connective tissue disorder.

## METHODS

### Patient inclusion and data collection

Data were prospectively collected on all patients undergoing hip arthroscopy between April 2009 and August 2014 and retrospectively reviewed. The inclusion criteria for this study were all patients who underwent arthroscopic LT reconstruction. The institutional review board approved this study. The exclusion criteria were patients with less than one year follow up.

#### Indications for LT reconstruction

The indications for LT reconstruction were patients who presented with pain and instability of the hip with an associated tear of the LT in the setting of generalized ligamentous laxity on a background of diagnosis of a known connective tissue disorder or a probable connective tissue disorder. LT tears were diagnosed with the aid of magnetic resonance imaging. Patients had persistent symptoms despite a minimum of 3 months of physical therapy. In this patient population, restoration of LT integrity was considered to an important adjunct to restoration of hip stability in addition to labral treatment and capsular plication, once bony morphology was determined as normal without any gross femur or acetabulum dysplastic characteristics.

#### Clinical evaluation

Symptoms of instability reported included the hip joint feeling unstable on either gait or range of movement, particularly extension and external rotation activities [28]. A history of instability of other joints was actively sought, as was a diagnosis of a connective tissue disorder [28]. Maximum flexion and maximum internal and external rotation at 90° of flexion were recorded. The specific test used to evaluate hip stability involved placing the patient in the supine position and the examiner placing the patient's hip in extension and external rotation. Discomfort or apprehension represented a positive finding. This implies abnormal physiologic motion resulting from soft-tissue deficiencies (e.g. anterior capsular laxity) [29]. Anterior, lateral and posterior impingement test signs were performed as described by Byrd *et al.* [30] and recorded as either present or absent. Ligamentous laxity was diagnosed according to Beighton's criteria [31].

### Imaging

#### Radiographic measurements

Radiographic views included an AP pelvic view, a 45° Dunn view and a false profile view. Measurements were made including the acetabular inclination (AI) angle using the method described by Jessel *et al.* [32], the lateral (LCEA) and anterior (ACEA) center edge angle of Wiberg [33], the presence of an ischial spine sign [34], crossover sign [34], alpha angle (Dunn view) [35] and femoral neck shaft angle. The crossover sign size was quantified according to its percent from the acetabulum diameter. All measurements were taken by the same orthopedic surgeon (X.X.) using a picture archiving and communication system computer program.

Preoperatively all patients underwent magnetic resonance imaging (MRI) using a 3T magnet of the affected hip to evaluate for intra-articular and extra-articular pathological abnormalities. The status of the LT was also documented.

### Surgical technique

**Fig. 1.**
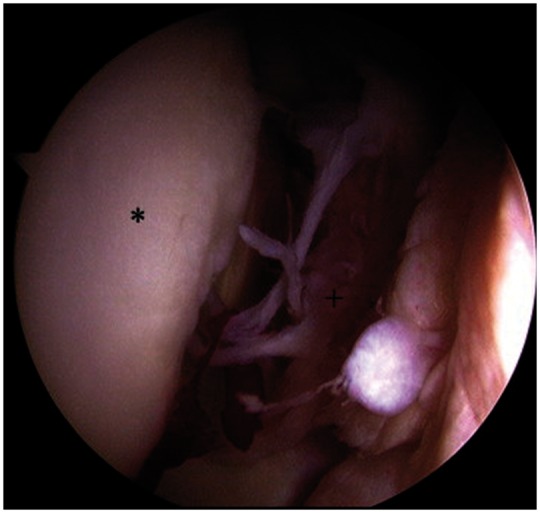
Intra-operative complete tear of the LT.

**Fig. 2.**
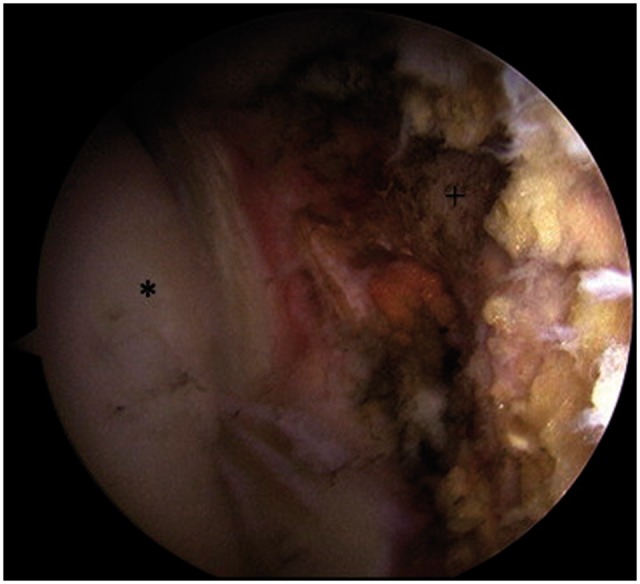
Appearance following debridement of the LT.

All hip arthroscopies were performed under general anesthesia in the supine position using a traction table and well-padded perineal post. All patients had muscle relaxant administered at the induction of anesthesia. Intra-operative diagnoses and procedures performed in the central, peripheral and peritrochanteric compartments were recorded. Labral tears were classified according to the Seldes classification [36]. A Seldes type 1 tear was disruption at the labral chondral junction and a Seldes type 2 tear was an intra-substance tear. The clock-face method was used to document the size and the location of the labral tear [37]. This method measures labral tearing using the 12-o’clock position as the most superolateral portion of the acetabulum and the 6-o’clock position as the transverse ligament. Disruption at the acetabular labral–chondral junction was described according to the acetabular labral articular disruption grading (ALAD) [14]. Chondral defects of the acetabulum and femur were graded according to the Outerbridge classification [38]. Any additional pathology in the joint was addressed before LT reconstruction. Bony pathology was corrected under fluoroscopic guidance. Acetabuloplasty was performed for pincer impingement, and a femoral osteoplasty was performed for cam impingement [39]. Full thickness articular cartilage damage was treated with debridement to create stable borders. Labral tears were treated with debridement or refixation. The decision on whether to debride or refixate the labrum in the setting of a labral tear depended on the stability of the labrum. Stable tears were debrided, whereas detached tears underwent repair. An iliopsoas fractional lengthening was performed by extending the medial capsulotomy and dividing the tendon at the level of the pelvic brim. The capsule was plicated using three or more stitches to create an inferior shift and imbrication in patients with generalized ligamentous laxity. The LT was examined and probed upon identification of a complete tear (Fig. 1) and classified as in Table I; the stump in the acetabular fossa was cleared with the Nav X ablation device (Arthrex, Naples, FL) and a shaver (Fig. 2).

**Table I.** LT classification

Type	Domb (%)	Villar
0	0	No tear
1	0–50	Complete rupture
2	50–100	Partial tear
3	100	Degenerative tear

#### Graft preparation

The graft choice may include a semitendinosus autograft or allograft or a tibialis anterior allograft. All grafts were double-stranded and secured at each end with a whip stitch using No. 2 fiberwire (Arthrex). The graft was then secured to a 12 mm RetroButton (Arthrex) with a 3 mm loop. The graft is prepared before tunnel preparation. The choice of autograft or allograft was dependent upon whether patients had undergone a previous procedure in which ipsilateral autograft was used or patient personal preference. The graft was sized to determine acetabular and femoral tunnel and reamer sizes. Typically the graft sizes were 7 or 8 mm.

#### Femoral and acetabular tunnels

**Fig. 3.**
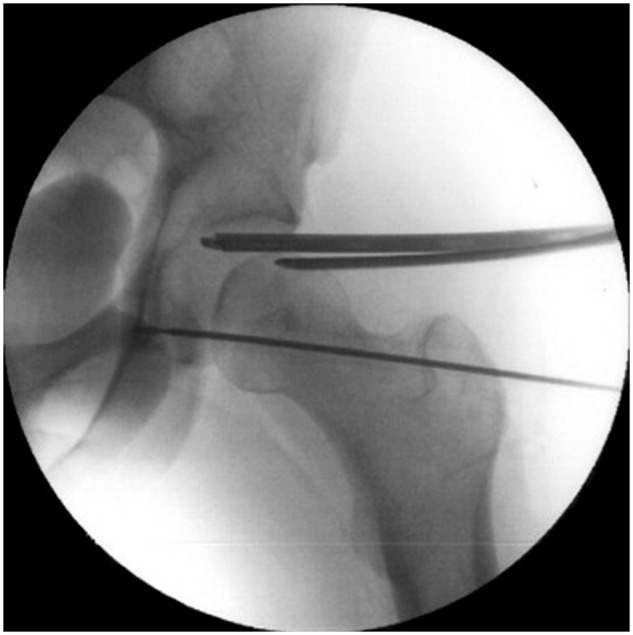
Fluroscopic guidance to determine tunnel placement for LT reconstruction.

A lateral 2 cm incision is made to approach the femoral transtrochanteric tunnel; the location is determined by fluoroscopy (Fig. 3). A 3.2 mm guidewire (Arthrex, cannulated reaming instruments) is passed through the lateral cortex of the greater trochanter, exiting through the center of the fovea in the footprint of the LT. This is performed using a “free-hand” technique with fluoroscopic assistance and direct visualization of the guide's exit point in the fovea. Over the guidewire, a cannulated reamer (Arthrex) is used to create the femoral tunnel. Drilling of the acetabular tunnel is performed through the femoral tunnel, with the hip internally rotated and abducted to achieve correct tunnel positioning in the cotyloid fossa. The anatomic insertion of the LT in the cotyloid fossa is made in the inferior portion of the fossa. To maintain a safe distance from the obturator vessels, the tunnel is placed slightly posterior to the center of the base of the fossa. preoperatively, potential vascular complications specific to this procedure are discussed in detail with the patient. Fluoroscopic assistance is used to ensure that the guidewire is not penetrating into the pelvis, and the drilling is performed cautiously to avoid plunging through the medial cortex of the acetabular fossa [20]. The tunnel passes through the medial wall with the same diameter.

#### Graft placement

Graft placement is performed by direct visualization and fluoroscopic assistance. Two knot-pushers are used to lead the graft–button complex through the tunnels; one knot-pusher is used to lead the button through the tunnel, and the second knot-pusher is used to flip the button over the medial cortex. Tunnel size did not have to be increased to accommodate passage of the knot pushers. Once the button has been flipped, tension is placed on the graft and fluoroscopy is used to ensure that the button has flipped and is secure.

**Fig. 4.**
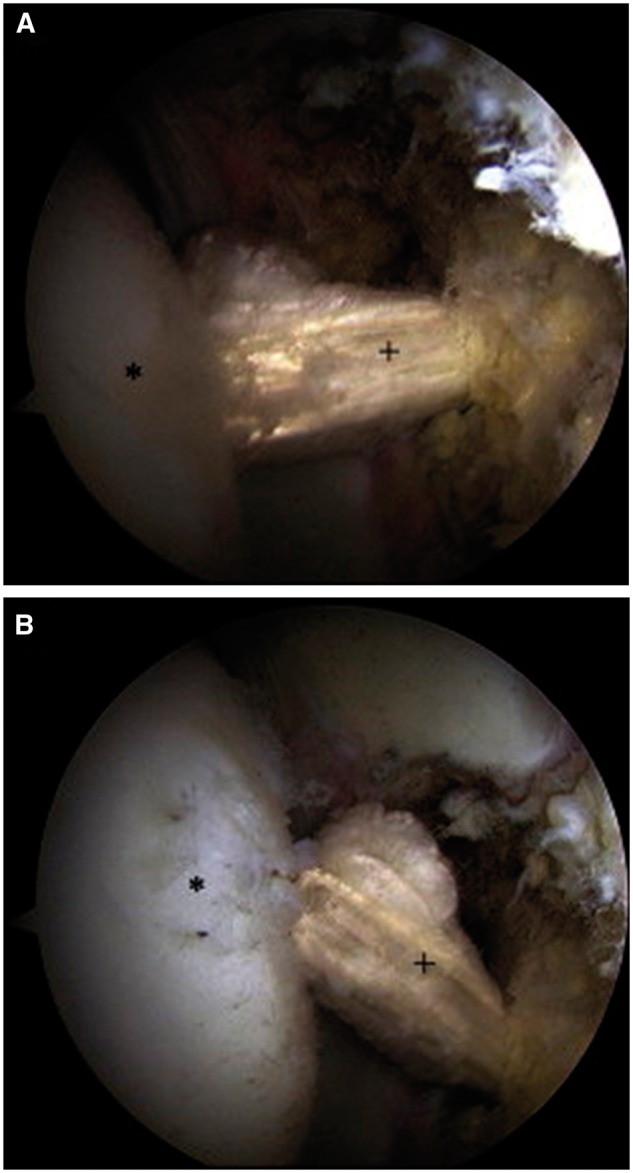
Reconstructed LT with a semitendinosis graft.

The motion and the tension of the graft are examined in internal and external rotation while the hip is in traction (Fig. 4). The traction of the leg is then removed while traction is maintained on the graft. The leg is positioned in 10° of hyperextension and 60° of external rotation, and a polyetheretherketone interference screw (Arthrex) is used for femoral fixation. The excess graft is cut flush with the lateral cortex of the femur followed by standard wound closure. The patient undergoes placement of an X-Act ROM hip brace (DJO Global, Vista, CA) and abduction pillow.

#### Rehabilitation and recovery

For the first 6 weeks, the patient is kept in a hip brace locked at 0°–90° of flexion at all times and is restricted to 20 lbs of foot-flat weight bearing. In addition, an abduction pillow is used at night for the same period. The patient starts physical therapy on the first post-operative day and is instructed to refrain from adduction and external rotation. Six weeks post-operatively, the use of the brace and crutches at six weeks discontinued and the patient continues physical therapy with an emphasis on strengthening the gluteus medius and core muscles, as well as gradual progression of range of motion.

### Outcome measures

Four hip specific outcome questionnaires were administered to patients pre- and post-operatively. These were the modified Harris Hip Score (mHHS), the non-arthritic hip score (NAHS) and the Hip Outcome Score—Sport-Specific Subscale (HOS—SSS). Patients were asked to estimate their pain on a visual analog scale (VAS) from 0 to 10, where 0 indicated no pain at all and 10 indicated the worse possible pain. These scores were recorded at the preoperative visit, at 3 months post-operatively and yearly thereafter. Patients rated their level of satisfaction after surgery on a scale of 0–10 with 10 being extremely satisfied and 0 being not satisfied at all. A satisfaction of 7 or more was considered a good/excellent result [40].

### Statistical analysis

The patients’ pre and post-operative results were compared using a 2-tailed Student's *t-test* for comparison of continuous variables and Chi-square test to compare categorical variable. *p*-values of <0.05 were considered statistically significant. Statistical analysis was performed with Microsoft Office Excel 2007 (Microsoft, Redmond, WA). Intra-rater reliability of radiographic measurements was determined using Bartko’s method for measuring a rater’s self-consistency [41] and was found to be greater than 0.79 for all measurements.

## RESULTS

### Demographics

During the study period, 2,463 hip arthroscopies were performed, of which 167 had complete tears of the LT. There were 487 partial tears treated, of which 97 were treated with debridement. Six reconstruction procedures were performed in five patients. Four reconstruction procedures in three patients had minimum one year follow up. The demographic findings are shown in Table II. The mean age of the cohort was 32.7 years. There were two females and one male. The mean body mass index (BMI) was 22.2 kg/m^2^. Mean follow-up was 21.4 (range 16.4–24.3) months. The decision to reconstruct the LT in these patients was based on all patients reporting symptoms that the hip felt unstable on gait and activities of daily living with a concomitant diagnosis of Ehlers-Danlos syndrome and a prior history of operative stabilization of another joint.

**Table II.** Demographics

Patient	Age (years)	Sex	BMI (kg/m^2^)	Follow-up (months)
1	22.6	M	25.9	24.3
2	21.3	F	22.2	27.8
3	43.5	F	20.4	17.3
4	43.7	F	20.4	16.4
Average	32.7	—	22.2	21.4

### Physical examination

The mean range of motion was flexion of 115° and, at 90° of hip flexion, internal rotation of 22.5° and external rotation of 50° (Table III). Three out of four, 0/4 and 4/4 of procedures had a positive anterior, posterior and lateral impingement tests respectively. All procedures developed apprehension with extension and external rotation of the hip. All patients had Beighton's scores of greater than 6.

**Table III.** Pre-operative clinical data

Patient	Flexion	IR (at 90° of flexion)	ER (at 90° of flexion)	Anterior Impingement	Posterior Impingement	Lateral Impingement	Apprehension with extension and external rotation
1	120	30	60	Y	N	Y	Y
2	120	40	50	N	N	Y	Y
3	100	10	45	Y	N	Y	Y
4	110	10	45	Y	N	Y	Y
Average	115	22.5	50	—	—	—	—

### Imaging

#### Plain imaging findings

With respect to indices for dysplasia, the mean LCEA was 30°, ACEA was 38° and AI was 1.5°. In one procedure, the acetabular floor was medial to the ilioischial line (i.e. profunda). One procedure had a prominent ischial spine and two procedures had positive cross-over signs. The neck shaft angle in two procedures was above 137° and less than 116° in one patient with bilateral reconstructions who had previous varus osteotomies (Table IV).

**Table IV.** Imaging findings

Patient	LCEA	ACEA	AI	Profunda	IS	Crossover	Neck shaft angle	Alpha angle	MRI LT tear
1	27	36	5	N	N	30	138	92	Full thickness
2	30	39	0	Y	Y	10	137	66	Full thickness
3	32	39	0	N	N	0	116	56	Full thickness
4	31	38	0	N	N	0	114	62	Partially torn/frayed
Average	30	38	1.25			10	126.5	69	

#### MRI findings

**Fig. 5.**
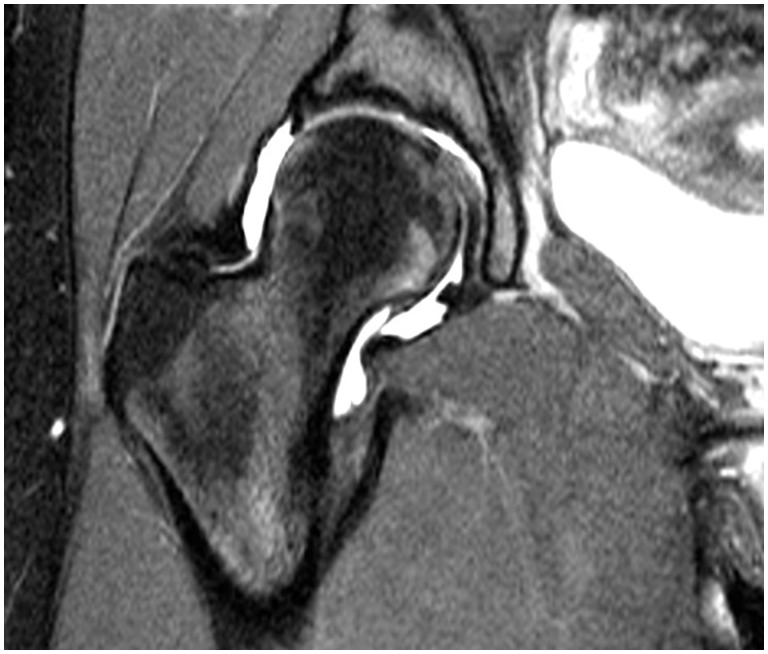
Appearance of an intact LT on MRI.

**Fig. 6.**
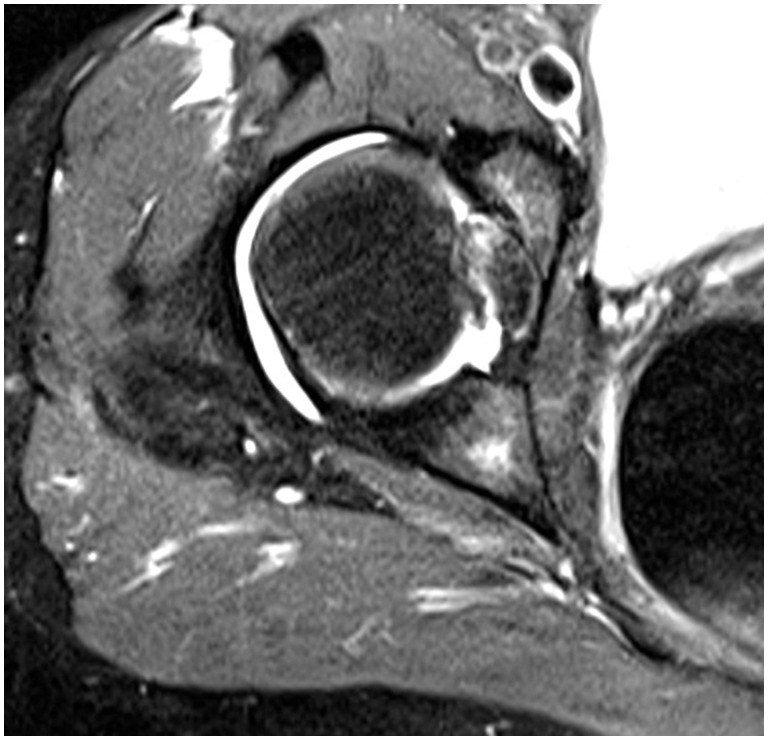
Appearance of a tear of LT on MRI.

Three procedures had full thickness tears of the LT on MRI and one procedure was considered to have partial. Fig. 5 shows the MRI findings of an intact LT with ligamentous connection between the fovea capitis and acetabular floor. Fig. 6 shows the findings of a partial or possible tear of the LT with increase signal within the ligament on T2 weighted imaging and bony edema within the region of the fovea capitis.

#### Intra-operative findings and concomitant procedures

Intraoperative findings and concomitant procedures are shown in Tables V and VI. Three out of four and 1/4 of procedures had an ALAD defect of grade 1 and 3, respectively. The size of the ALAD defects ranged from 1 to 1.5 cm^2^. LT tears were graded as a Domb 3 and Villar 1 in 3/4 of procedures and as a Domb 2 and Villar 2 in 1/4 of procedures. Two procedures had a LT reconstruction using semitendinosis autograft and two procedures’ patients had tibialis anterior allografts.

**Table V.** Intra-operative findings

Patient	Seldes tear	Location of tear	Size of tear	ALAD type	ALAD size (cm^2^)	Femoral outerbridge	Outerbridge size (cm^2^)	LT tear domb	LT tear villar
1	1	13.5–14.5	1 h	1-Softening	1	0	—	3	1
2	—	—	—	1-Softening	1.5	0	—	3	1
3	—	—	—	3-Fissuring	1	0	—	3	1
4	1&2	12.0–15.0	3 h	1-Softening	1	0	—	2	2

**Table VI.** Concomitant procedures

Patient	Labral treatment	Capsular treatment	Cartilage	Iliopsoas
1	Repair	Plication	None	None
2	None	Plication	None	None
3	None	Plication	None	None
4	Repair	Plication	None	None

#### Patient reported outcomes

Apart from one procedure not competing a HOS—SSS score, all procedures had pre-operative and postoperative PRO outcomes scores recorded (Table VII). The average preoperative score for mHHS, NAHS and HOS—SSS were 42.6, 44.1, and 25.8 respectively. In three out of the four procedures, there was an improvement in all PROs except VAS, which worsened by two points in one procedure. For the other procedure, there was a worsening of all PROs.

**Table VII.** Outcomes

		Pre-Operative				Follow Up						Delta			
Patient	Follo-up (months)	mHHS	HOS-SSS	NAHS	VAS	mHHS	HOS-SSS	NAHS	VAS	Satisfaction	Time	mHHS	HOS-SSS	NAHS	VAS
1	24.3	21	NA	54	10	64	NA	56.25	3	10	24.03	43	NA	2.25	−7
2	27.8	70	42	71	1	77	44.44	80	3	8	24.15	7	2.44	9	2
3	16.4	36.26	3.125	27.5	8	90.1	34.38	50	0	10	12.64	53.84	31.255	22.5	−8
4	17.3	43	38	24	10	38.46	25	25	10	0	13.85	−4.54	−13	1	0
Average	21.5	42.6	20.8	44.1	7.3	63.4	25.9	52.8	4	7	18.7	24.8	5.1	8.7	−3.3

## DISCUSSION

The purpose of this study was to report on the results of four LT reconstructions in three patients. Two of the patients were female. All patients had a positive lateral impingement sign. There was no pattern of radiological findings with respect to acetabular coverage, version and depth. Excluding the patient that had previous varus femoral osteotomies, the remaining two patients had a valgus neck shaft angle. Intra-operatively, there was no association between LT tears and labral and chondral pathology grade. Capsular plication was concomitantly performed in all patients. With respect to PROs, three out of four procedures demonstrated an improvement in all four PROs, except for one procedure that had a worsened VAS. In one patient, there was a decline in PRO scores and that patient subsequently underwent revision reconstruction.

A recent review of management of LT tears concluded that efforts needed to be concentrated on refining the physical examination and imaging criteria for better detection preoperatively [17]. In our cohort of four patients, there was a wide variation in hip range of motion and presence of impingement signs with the exception of lateral impingement. The recently reported LT test, with a sensitivity and specificity of 90% and 85%, respectively, may help in clinical assessment [42]. In 3/4 of patients, a definite tear of the LT was detected on MRI. Bryd and Jones [15] reported a low sensitivity for radiological detection of LT pathology. They reported that only two diagnoses were made among 37 patients who underwent MR arthrogram, computed tomography or bone scanning for LT pathologic conditions.

In three out of four procedures, there was a definite improvement in PROs. A patient who had bilateral reconstructions had a poor outcome on her right side. This patient was a 43-year-old female who presented with bilateral hip pain and instability for 3 months. She had been diagnosed with hyper-mobility secondary to Ehlers–Danlos Syndrome and had bilateral femoral osteotomies at the age of 15 to reduce anterior instability caused by excessive femoral anteversion. She had a reduction in her mHHS and HOS-SSS as well as maintenance of her VAS score of 10 on the right. Conversely, her left hip had an excellent outcome with a 53 point improvement in her mHHS and a 0 VAS score at 1 year follow-up. No obvious cause for the discrepancy was found but the patient subsequently underwent a right revision reconstruction and open capsular reconstruction for recurrent posterior instability for which the 3-month follow-up results are pending. The remaining patients all had an improvement in PROs with satisfaction ranging between 7 and 10.

There are three studies that have reported on the results of LT reconstruction [20, 26, 27]. Two of the studies are case reports and one study is a case series of four patients. Grafts used in these studies include a synthetic knee medial collateral ligament; a double-stranded semitendinosus autograft and an iliotibial band tendon autograft. Philippon *et al.* [26] had a 2-year follow-up in two out of four patients in which one did not have preoperative scores and other patient had an improvement of mHHS of 7 points at 2 years and 21 points at 3 years. Amenabar *et al.* [27] published a case report in which at 12 months, the patient’s mHHS and NAHS improved by 47 and 22 points, respectively. These reports are in concordance with our results.

One strength of this study was that it has the largest number of patients with minimum 1-year follow-up. Furthermore, the study incorporated three PROs as it has been reported that no single PRO provides a comprehensive assessment post hip arthroscopy [43]. The clinical relevance of the present study’s findings is that in patients with hip instability with a background of ligamentous laxity and multi-joint instability due to a connective disorder, LT reconstruction associated with other soft tissue stabilization is associated with improved outcomes.

## LIMITATIONS

The study had limitations. Despite having the largest number of cases of LT reconstructions, the number of patients in the case series was small, limiting the statistical significance of findings. Specific clinical tests for LT pathology were not included because tests had not been validated or published at the time of assessment. There was no control arm and, therefore, it is difficult to know to what extent LT reconstruction caused an improvement in PROs in the setting of other concomitant procedures. In addition, there has been no mechanical testing to determine the acetabular graft contact area with this reconstruction technique or whether the graft bonds with the tunnel wall or remains suspended from the retrobutton. Furthermore, all patients did not undergo follow-up MRI imaging to determine the integrity of the reconstruction. Finally, graft choice, particularly autologous graft, may have potentially been an issue in this patient population with a background history of abnormal collagen. However, in the patient population with hip instability due to an underlying connective tissue abnormality, this study demonstrates that LT reconstruction with concomitant labral treatments and capsular plication improved symptoms. The study adds to the body of knowledge on the evolving indications of LT reconstruction allowing future research to compare reconstruction techniques including methods of fixation.

## FUNDING

This work was supported by the American Hip Institute, which receives general research support from Arthrex, Stryker, Breg, ATI, and Pacira. No funding was received directly for this work.

## CONFLICT OF INTEREST STATEMENT

The senior author is a board member at the American Hip Institute and AANA Learning Center Committee; a paid consultant for Arthrex, Pacira, Stryker, and Amplitude; and receives royalties from Arthrex, Orthomerica, and DJO Global.
